# Influence of Silver Addition on Structure, Martensite Transformations and Mechanical Properties of TiNi–Ag Alloy Wires for Biomedical Application

**DOI:** 10.3390/ma13214721

**Published:** 2020-10-22

**Authors:** Gulsharat Baigonakova, Ekaterina Marchenko, Timofey Chekalkin, Ji-hoon Kang, Sabine Weiss, Aleksei Obrosov

**Affiliations:** 1 Laboratory of Medical Alloys and Shape Memory Implants, Tomsk State University, 634045 Tomsk, Russia; chaaarmy@mail.ru (G.B.); marchenko84@vtomsk.ru (E.M.); 2 TiNiKo Co., R&D Center, Ochang 28119, Korea; hunywell@gmail.com; 3 Department of Physical Metallurgy and Materials Technology, Brandenburg University of Technology, 03044 Cottbus, Germany; weiss@b-tu.de (S.W.); aleksei.obrosov@b-tu.de (A.O.)

**Keywords:** shape memory alloy, silver, doping, mechanical properties, nanostructured material, wire

## Abstract

The microstructural and functional behavior of TiNi-based wires with a silver content of 0–1.5 at.% was evaluated. The concentration range for Ag doping determined for the TiNi wires with potential for the medical industry was 0–0.2 at.%. Microstructure analysis of TiNi wires with different silver contents at room temperature indicated a multiphase structural state. Various internal structures with tangled grain boundaries were formed by intense plastic deformation. The nanocrystalline structure and phase state of wire with the minimum silver content (0.1 at.% Ag) provide full shape recovery, the greatest reversible strain, and optimal strength and ductility. TiNi ingots with a high Ag content (0.5–1.5 at.%) cracked under minimum load due to excess silver that crystallized along the grain boundaries and broke cohesion bonds between the TiNi grains.

## 1. Introduction

The development and study of new TiNi-based materials is a promising direction in the field of medical materials science. The special properties of the alloys are shape-memory and superelasticity due to reversible austenite-martensite transformations caused by changes in external conditions such as load and temperature [1,2,3,4]. There are differences between thermal and stress-induced martensite transformations that affect the magnitude of the transformation strain [5,6].

TiNi-based wire is widely used in the manufacture of both temporary and long-functioning surgical implants because of bio-inertness and ability to undergo viscoelastic deformation with tissues, surviving millions of deformation cycles without failure [7,8]. In order to increase the survival rate of TiNi implants, an additional antibacterial effect is required. This effect can be achieved with silver doping [9,10]. At the same time, it is important to obtain a technological alloy that can be used to produce implants with high functional properties including fully reversible deformation, high strength, and ductility.

Nowadays, doping and coating of coarse-grained TiNi alloys with Ag with a concentration of 0.5 to 9 at.% without subsequent thermomechanical treatments is a common practice worldwide [11,12,13,14,15]. In terms of using TiNi–Ag materials in the medical industry, alloying TiNi with Ag provides the alloy with new attributes suitable for biomedical applications, improving its cytocompatibility and antibacterial capacity [11], while increasing yield and tensile strengths [16,17]. TiNi composite surface films doped with silver (4–10 at.%) enhance mechanical strength, biocompatibility, and corrosion resistance to implants [18,19,20]. However, as the silver concentration exceeds 5 at.%, the antibacterial properties of the alloy deteriorate. Therefore, doping with lower silver concentration is of particular interest. The shape memory effects of Ag-doped TiNi were studied at a concentration of 1.4 at.% silver [12], and martensite transformations as well as microhardness were mainly considered in the range of 0.6–1.9 at.% Ag [21,22,23,24]. However, the issue of how lower silver content (< 0.5 at.%) could affect the functional characteristics and deformability of TiNi–Ag wires has not yet been studied thoroughly. 

The above-mentioned overview of the effect of high silver doping on the structure and properties of TiNi indicates the relevance of this issue for biomedical applications. During implant manufacturing, alloys are subjected to numerous thermo-deformation effects, so deformation properties are of high importance. At the first stage, the structure, shape memory, as well as mechanical and biocompatibility properties of TiNi-based alloys with 0–1.5 at.% Ag were studied before thermo-mechanical treatments [25,26]. Interpretation of the resulting dependencies between phase composition, microstructure, functional and mechanical properties served as the scientific basis for creating new implants from TiNi wires with enhanced performance. 

This study is the second stage of research aimed at creating biocompatible TiNi alloys with high functional properties and an additional antibacterial effect. The purpose of the current study is to determine the range of silver content for the production of TiNi–Ag wires obtained by intense plastic deformation and to study their structure, phase composition, shape memory effect and mechanical properties. 

## 2. Materials and Methods 

TiNi–Ag alloys were melted in an induction furnace according to the following doping scheme: Ti_50_Ni_50−X_Ag (X = 0, 0.1, 0.2, 0.5, 1, and 1.5 at.%). The combined method of intensive plastic deformation, consisting of multiple processes of cold rolling and intermediate annealing (T = 400–450 °C), was used to produce wire with a diameter of 1 mm. Three main production stages were used: i) rolling of ingots (8 cycles) up to a bar 80 mm thick; ii) rotary-forging of round bars up to a 3.5 mm wire (7 cycles); iii) hot wire drawing up to 1 mm (25 cycles). The following requirements of cold deformation processes were fulfilled: low degree of compression per cycle, and intermediate annealing to reduce work-hardening effects and increase metal ductility. Intermediate annealing temperatures were selected in such a way that the recrystallization of grains occurred, but there was no increase in grain size.

**Figure 1:**
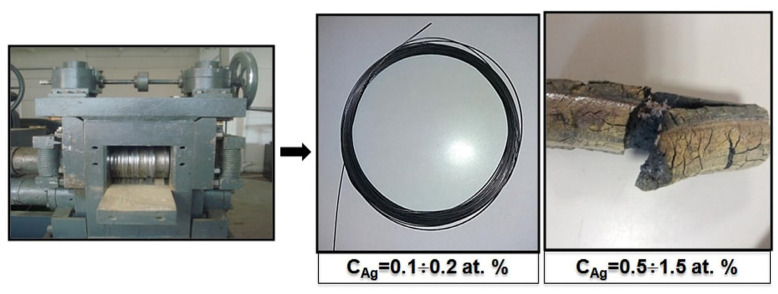
Rolling and drawing of TiNi–Ag with different silver content.

Ingots with a high Ag content (0.5–1.5 at.%) cracked under minimum compression using a rolling mill (Figure 1).

The phase composition and structural parameters of the phases were studied using a Shimadzu XRD-6000 diffractometer (Kyoto, Japan) with CuKα radiation. The diffraction patterns were indicated by means of the PowderCell 2.4 full-profile analysis program and compared with the PDF 4+ database. A survey of the side surface of wires in the direct scan geometry θ/2θ did not give the expected results. The X-ray profiles exhibited only reflections of titanium oxides, carbides, and nitrides because of a screening effect of the surface oxide layer on the matrix. Therefore, a special method of sample preparation was developed for the examination of the matrix of 1 mm thick wire. In order to obtain a flat surface, 20 wire segments were connected with a thin TiNi thread into a dense “bundle” and filled with epoxy resin at the base. After that, the end surface of the “bundle” was mirror polished. The shape memory effect parameters were studied in tension at a constant load of 2 kg. Transmission electron microscopy (TEM) imaging of thin foils were performed using a JEM-2100 transmission electron microscope (JEOL, Tokyo, Japan) at an accelerated voltage of 200 kV. The mechanical properties were studied using an Instron testing machine under tensile strain conditions.

## 3. Results and Discussion

Ingots with a high Ag content (0.5–1.5 at.%) cracked under minimum compression, as depicted in Figure 1. The deformation process is significantly affected by the grain/grain boundary interface interaction, since grain boundaries have a chemical composition and characteristics that differ from those of the grains themselves [27,28]. The grain boundaries have a significant impact on the deformation conditions, since their chemical composition and properties differ from those of the grains. There are zones at the grain boundaries that prevent deformation. The movement of atoms in the grain boundaries required for intergranular deformation is complicated by the presence of insoluble Ag phases. Silver phases in TiNi alloys with an Ag concentration of more than 0.5. at.% were formed as a result of their limited solubility in TiNi [29]. The liquation of excess silver along the grain boundaries in the alloys broke cohesion bonds between TiNi grains. Thus, the presence of phases along the grain boundaries changes the deformation mechanism; as deformation is developing, cavities arise in the form of microcracks and micropores, which lead to cracks nucleating at specific points and propagating across the material.

**Figure 2:**
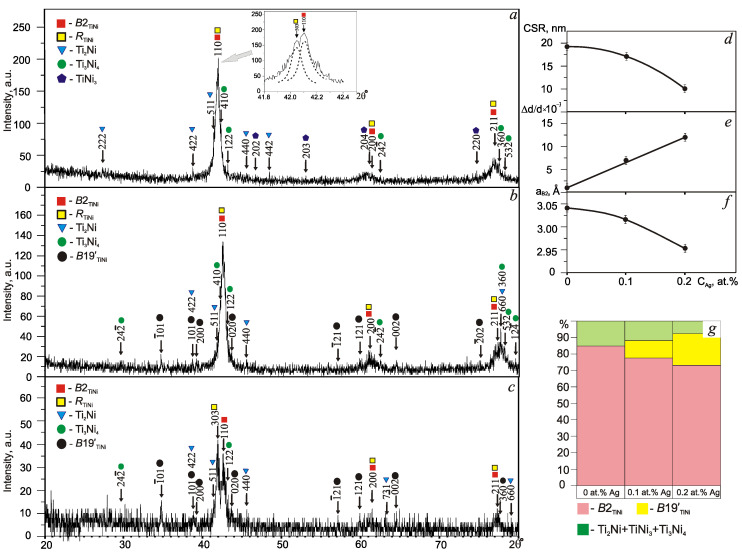
XRD patterns of TiNi wires with silver concentrations of 0 (**a**), 0.1 (**b**), and 0.2 at.% (**c**); concentration dependences in the parent phase on: coherent scattering regions (**d**), internal stresses (**e**), lattice parameter (**f**), phase volume fraction (**g**).

XRD analysis showed that TiNi wires with different silver concentrations at room temperature are characterized by a multiphase structural state (Figure 2). The purity of the silver used as a dopant was 99.9%, as confirmed by a certificate. However, as is evident from the presented XRD patterns, no traces of Ag-related phases were revealed. The findings on elemental mapping reported in our previous work have also proved no aggregate of metallic Ag0 or oxides Ag_x_O_y_ [25]. The main structural component of the wires is intermetallic TiNi in *B*2 and *R* modifications. This is confirmed by the angular distribution and high intensity of the main reflections of these phases. In the angular interval, 2θ = 42–43°, a pronounced splitting of the main reflection of the *B*2 phase with a partial overlap of {110}*_B_*_2_ and {303}*_R_* peaks was found (inset in Figure 2a). These results correlate with the electrical resistivity ρ(T) data in the same series of samples [25].

The sizes of the coherent scattering regions (CSR) of the *B*2 phase in the studied wires do not exceed 30 nm, which indicates a fine-crystalline structural state after multiple mechanical and thermal effects (Figure 2d). Qualitative XRD analysis revealed the presence of structural reflections from TiNi phases with a stoichiometry of Ti_2_Ni, Ti_3_Ni_4_, and TiNi_3_ with a total volume fraction not exceeding 15 vol.%, which were distributed differently depending on the composition. The occurrence of Ni-rich phases is stemmed from the segregation of Ti to the alloy surface during drawing, with subsequent formation of an oxide layer through intermediate annealing [30,31]. No phases with Ag were detected in the XRD diffraction pattern of wire samples. A small amount of silver is not sufficient for the formation of individual intermetallic compounds; therefore, the silver completely dissolves in the matrix replacing titanium in its sublattice, as indicated by a decrease in the lattice parameter (Figure 2e).

Silver dissolves in the TiNi*_B_*_2_ phase to a limited extent, up to 0.26 at.% [11,12]. The addition of 0.1 at.% Ag causes the occurrence of reflections of the *B*19’ phase. An increase in silver content in combination with intense plastic deformation leads to an increase in the preferential orientation of the crystal lattice of *B*2-phase grains in the {200} direction from 17 to 35%. 

An increase in the concentration of Ag in TiNi solid solution decreases the *B*2 lattice parameter, thereby changing interatomic interaction forces and increasing internal elastic stresses Δd/d from 1.2 × 10^−3^ to 12.45 × 10^−3^ (Figure 2f), provoking a *B*2→*R*→*B*19′ phase transformation. The volume fraction of the peritectic Ti_2_Ni phase decreases from 7 to 4 vol.%, and the amount of the metastable phase Ti_3_Ni_4_ increases from 5 to 8 vol.%.

Qualitative XRD analysis of the diffraction pattern of the wire with 0.2 at.% Ag showed a high content of *B*19’ martensite and Ni-rich fine phase Ti_3_Ni_4_, and structural lines from the secondary Ti_2_Ni and Ti_3_Ni_4_ phases that were less intense than those of the wire with 0.1 at.% Ag. Assessment of the volume fractions of phases showed that when the silver concentration increases, the volume fraction of *B*2 austenite and secondary phase Ti_2_Ni decreases, while that of Ti_3_Ni_4_+TiNi_3_ increases (Figure 2g).

**Figure 3:**
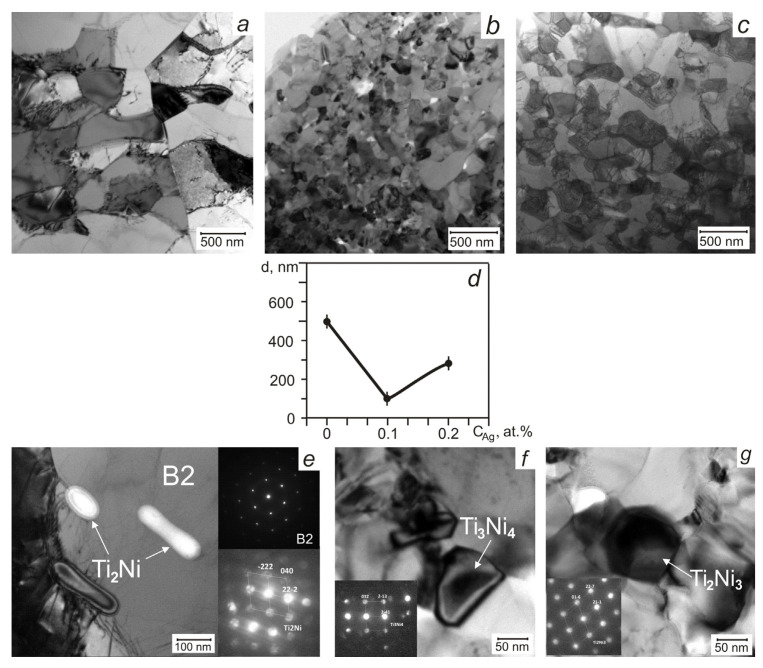
Transmission electron microscopic (TEM) images of TiNi wires with different Ag concentrations: 0 at.% (**a**), 0.1 at.% (**b**), 0.2 at.% (**c**); concentration dependence of the average grain size (**d**); TEM image of the parent and Ti_2_Ni phase, Ti_3_Ni_4_, Ti_2_Ni_3_ precipitations with corresponding electron diffraction patterns (EDP) presenting in the studied wires (**e–g**).

The average grain size and microstructural features evident from Figure 3 indicate that in alloys with different silver contents, the intense plastic deformation formed various internal structures where the average grain size changed nonlinearly. The small addition of Ag has an ambiguous effect on the processes of relaxation of internal stresses and dynamic recrystallization, variously inhibiting or inducing them. Figure 3 shows that the grain size and the number of deformation defects at the boundaries and in the grain volumes are different, and phases of different sizes are formed.

**Figure 4:**
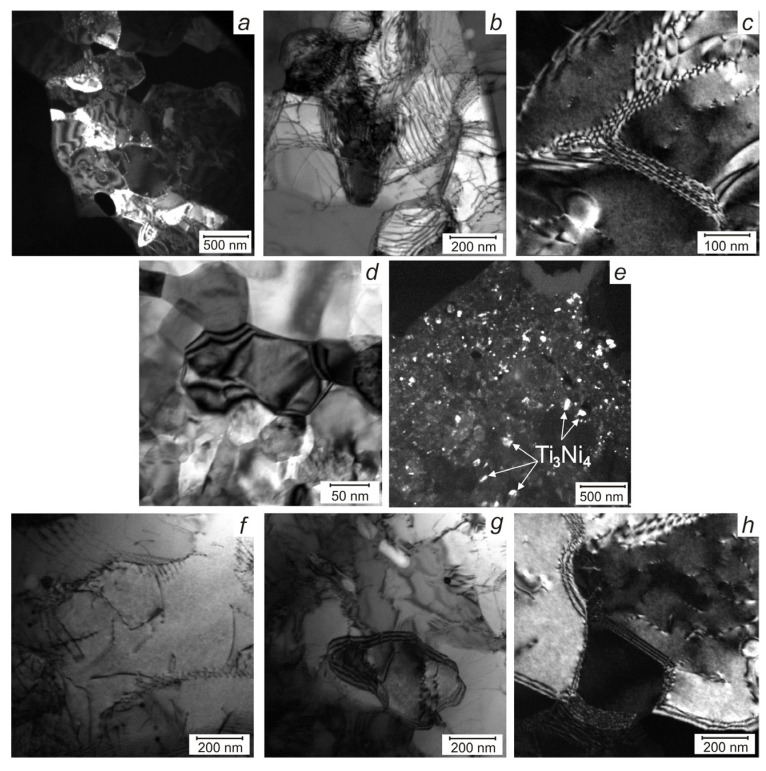
TEM images of areas with high curvature of the crystal lattice and dislocations of TiNi wire (**a–c**); areas with inhomogeneous contrast of displacement bands and with uniformly distributed finely dispersed T_3_Ni_4_ phases of TiNi–Ag_0.1_ wire (**d,e**); areas with dislocation defects of TiNi–Ag_0.2_ wire (**f–h**).

The TiNi wire without Ag is characterized by a fully crystalline ultrafine-grained structure with high dislocation density and an average size of recrystallized grains of 500 ± 50 nm (Figure 3 and Figure 4). In Figure 4a–c inhomogeneous contrast of the extinction contour in the subgrains indicates the presence of a substructure in which the curvature of the crystal lattice, level of stresses, and density of dislocations is high. The lamella TEM image with the corresponding electron diffraction patterns (EDP) enabled to identify of the TiNi_B2_ matrix, white elongated Ti_2_Ni inclusions 50–150 nm in size along the grain boundaries, and fine Ni-rich Ti_3_Ni_4_ precipitates 20–60 nm in size in individual areas of the matrix.

In the TiNi wire with 0.1 at.% Ag, a more homogeneous nanocrystalline structure was formed without dislocation defects and with an average grain size of 100 ± 50 nm. No dislocation defects were detected in the grains because the small grain sizes prevent the development of dislocation slip. There are areas with inhomogeneous contrast of displacement bands types because of intergranular stresses near the boundaries, caused by the grain orientation difference on the boundary (Figure 4d). EDP identification from different areas of the matrix showed the presence of parent *B*2, martensite *R* and *B*19’ phases, Ti_2_Ni phase with sizes of 10–50 nm, and Ti_3_Ni_4_ and Ti_2_Ni_3_ phases (10–30 nm). Finely dispersed Ti_3_Ni_4_ precipitates are uniformly dispersed throughout the volume (Figure 4e). The total number of Ti_2_Ni particles along with the TiNi grain boundaries was half of the precursor TiNi wire, which is in good agreement with the XRD data.

In the TiNi wire doped with 0.2 at.% Ag, an ultrafine-grained structure with dislocation defects similar to that of the TiNi wire (Figure 4f–h) and average grain size of 250–300 nm (Figure 3c) was observed. Identification of diffraction patterns from individual wire areas confirms the presence of the parent *B*2 and *R* phases, and secondary Ti_2_Ni and Ti_3_Ni_4_ ones. No diffraction reflections from martensite B19’ were found in the investigated lamella, although the volume fraction of this phase in the XRD spectrum attained 18 vol.%. Figure 3e–g indicates the EDP for typical *B*2, Ti_2_Ni, Ti_2_Ni_3_, and Ti_3_Ni_4_ phases persist in the wires studied.

The formation of ultrafine-grained and nanocrystalline structures is a result of thermomechanical treatment by rolling and drawing with intermediate annealing [7,32] during which a number of excess equilibrium TiNi_3_ and metastable Ti_2_Ni_3_ and Ti_3_Ni_4_ phases are precipitated [33,34,35]. The structures formed have a positive effect on the resistance of the material to plastic deformation. 

The functional properties significantly depend on the microstructural features, including grain size, defects, precipitated secondary phases, and internal stress, which form during intense plastic deformation in the process of obtaining TiNi–Ag wire.

**Figure 5:**
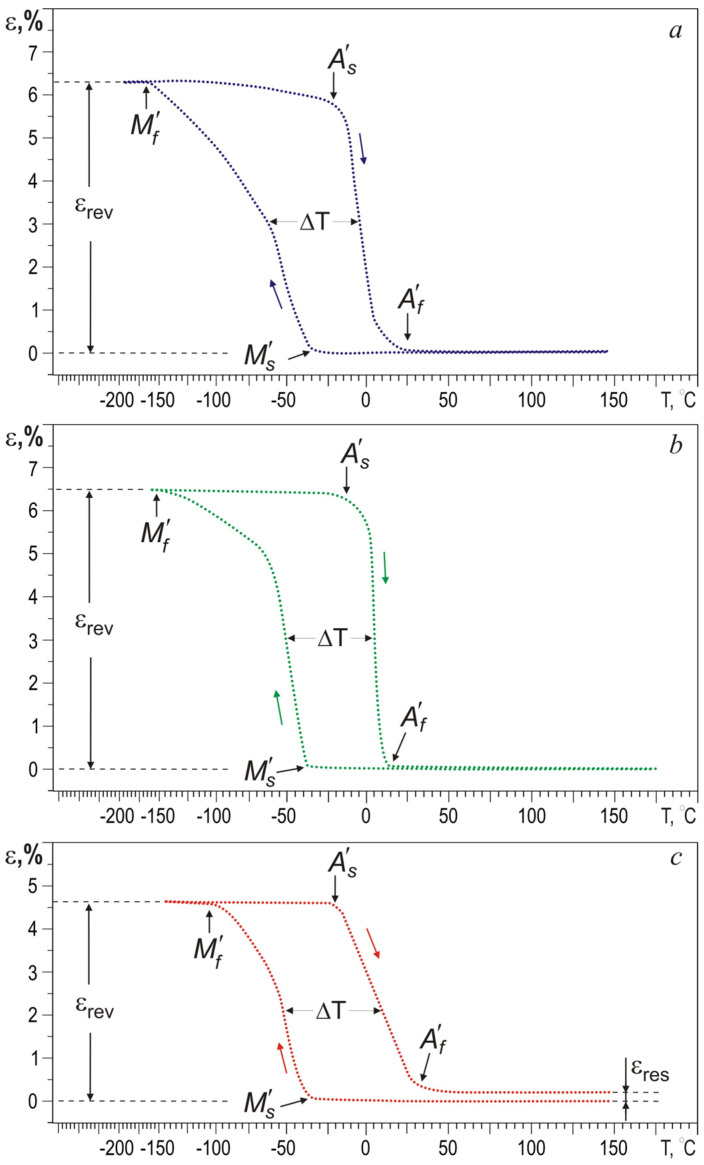
Experimental curves in terms of ε-T of TiNi wires with different content of Ag: 0 (**a**), 0.1 (**b**), and 0.2 (**c**) at.%.

Figure 5 shows the features of the shape memory effect (SME) for all wire samples; and the measured SME parameters are given in Table 1. In the cooling-heating cycle under a constant tensile load, all wires are characterized by high reversible deformation values. However, in the wire with 0.2 at.% Ag, the defect structure of the *B*2 phase has suppressed the martensite transformation due to an increase in the Ag concentration in the TiNi solid solution and the presence of a high density of dislocations as a result of intense plastic deformation.

The onset temperature of direct martensite transformation (MT) insignificantly depends on the silver concentration and remains stable at 38 ± 2 °C. This means that the crystal lattice of the *B*2 phase is stable to martensite transformations at the macro level under load despite a change in silver concentration. It is known that doping of TiNi alloys with a third element leads to a sharp decrease in the MT temperature or complete suppression, especially under conditions of intense plastic deformation. However, noble elements (palladium, platinum etc.) at low concentrations form solid solutions based on the *B*2 structure and have contrary effects on the stability of *B*2 austenite [36], as in our case with silver [17]. The thermal hysteresis Δ*T*, which characterizes the amount of energy dissipation during the phase transformation, increases from 55 to 68 °C with a silver concentration increase in the 0–0.2 at.% range.

The main contribution to the width of thermal hysteresis is made by incoherent particles of the Ni-rich secondary phases, in which energy dissipation occurs during the movement of martensite boundaries in shape recovery. 

In wires with 0.1 and 0.2 at.% Ag, during MT under loading with decreasing temperature, leads to the formation of dislocation defects during the movement of the austenite-martensite (A→M) interfaces. Then, upon heating during reverse MT, these defects impede the movement of the M→A interface. This phenomenon leads to a broadening of the temperature range of the reverse MT under load, as also reported in [37,38]. 

The characteristic temperatures *M*’*_f_*, *A*’*_s_*, and *A*’*_f_* decreased, as residual strain occurs in TiNi wires with 0.2 at.% Ag. The residual strain is caused both by the accumulation of the plastic strain component and by defects that arise during intense plastic deformation. The plastic strain component is the main mechanism for the dissipation of elastic energy. These phenomena significantly affect the movement of interfaces and lead to irreversible effects. The residual strain appears because of the irreversible plastic deformation caused by the high content of finely dispersed Ti_3_Ni_4_ precipitates, which do not undergo martensite transformations. The slope of the thermal hysteresis loop is related to the high heterogeneity of the structure and the density of defects in the initial TiNi wire and in the TiNi wire with 0.2 at.% Ag, and is in good agreement with TEM studies.

**Figure 6:**
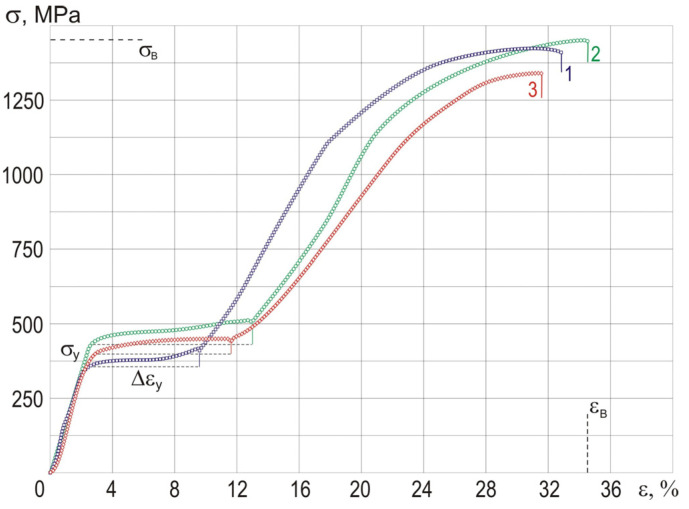
Stress-strain curves of TiNi–Ag wires with different Ag content: 0 (**1**), 0.1 (**2**), and 0.2 (**3**) at.%.

Figure 6 shows the mechanical behavior of the wires during tensile tests. Tensile tests performed at room temperature show that *B*2→*R*→*B*19′ martensite transformation caused by deformation occurs in all wires, as evidenced by the phase yield region Δε_y_. The linear section depicted on the σ-ε curves corresponds to the strain accumulation of the *B*2 phase. The presence of a stress plateau indicates the development of the stress-induced *B*2→*R*→*B*19′ martensite transformation and an increase in the volume fraction of the *B*19′ phase [39]. A comparative analysis of stress–strain diagrams demonstrates the effect of silver content on the length of the phase yield section Δε_y_, tensile strength σ_в_, and maximum strain before fracture ε_в_ (Table 2). The maximum values of the Δε_y_, σ_в_, and ε_в_ in the stress–strain curves belong to the TiNi wire doped with 0.1 at.% Ag.

The homogeneous nanocrystalline dislocation-free structure with a significant volume fraction of finely dispersed reinforcing Ti_3_Ni_4_ precipitates is the rationale for the high strength and ductility of the TiNi wire doped with 0.1 at.% Ag. In addition, with increased density of grain boundaries, the uniformity of plastic strain in the material volume increases, and the internal stress level decreases. The lower mechanical properties of other wires are associated with a high density of dislocations, which are stress concentrators and can cause destruction [38,39]. The presence of a large volume fraction of incoherent Ti_2_Ni particles in the TiNi wire and their interaction with the dislocation substructure also lead to a strong stress concentration and make the wires less durable.

## 4. Conclusions

Ultimately, the concentration range for Ag microalloying was found for TiNi wires (0–0.2 at.%) obtained by intense plastic deformation. The combined method of intense plastic deformation, consisting of multiple processes of cold rolling and intermediate annealing (T = 300–450 °C), was used to produce wire with a diameter of 1 mm. The optimal balance between strength, ductility, and shape memory parameters was achieved at a silver concentration of 0.1 at.%. The mechano-thermal treatment promotes the formation of a submicrocrystalline structure and recrystallization of the finely dispersed phases TiNi_3_, Ti_2_Ni_3_ and Ti_3_Ni_4_, and reduces the size of Ti_2_Ni particles. In the wires, the minimum silver concentration (0.1 at.% Ag) provides full recovery of the shape (ε_res_ = 0%), the greatest reversible strain (ε_rev_ = 6.5%), and optimal strength (σ_в_ = 1450 MPa) and ductility (ε_в_ = 34.4%) properties. This is achieved by virtue of the dislocation-free homogeneous nanocrystalline structure with an average grain size of 100 ± 50 nm and a high concentration of Ti_3_Ni_4_ dispersion-strengthening precipitates in the TiNi(Ag) matrix. TiNi wire 1 mm in diameter and doped with 0.1 at.% silver may be a suitable alternative to implants of known titanium alloys for use as actively functioning implants in biomedical applications.

## Figures and Tables

**Table 1:** Comparative shape memory effect (SME) parameters in TiNi and TiNi–Ag wires.

	*M*’*_s_*, °C	*M*’*_f_*, °C	*A*’*_s_*, °C	*A*’*_f_*, °C	Δ*T*, deg	ε_rev_, %	ε_res_, %
TiNi	−36	−164	−22	25	55	6.2	0
TiNi_49.9_–Ag_0.1_	−40	−145	−14	13	60	6.5	0
TiNi_49.8_–Ag_0.2_	−38	−108	−27	27	68	4.7	0.2

**Table 2:** Mechanical properties of the TiNi–Ag wires.

	σ_y_, MPa	σ_в_, MPa	Δε_y_, %	ε_в_, %
TiNi	350 ± 20	1370 ± 20	7 ± 0.5	32 ± 0.5
TiNi_49.9_–Ag_0.1_	435 ± 20	1450 ± 20	10 ± 0.5	34 ± 0.5
TiNi_49.8_–Ag_0.2_	400 ± 20	1330 ± 20	8 ± 0.5	31 ± 0.5
